# Socioecological analysis of determinants of early adolescent sexuality in Benin: a qualitative study

**DOI:** 10.11604/pamj.2023.44.137.35978

**Published:** 2023-03-17

**Authors:** Bankole Murielle Sonia, Patrice Ngangue, Bationo Nestor, Soubeiga Dieudonné, Ahounou Adnette, Philibert Leonel, Birama Apho Ly

**Affiliations:** 1 Institut de Formation et de Recherche Interdisciplinaires en Sciences de la Santé et de l´Education (IFRISSE), Ouagadougou, Burkina Faso,; 2 Institut National Medico-Sanitaire, Université d´Abomey Calavi, Cotonou, Benin,; 3 Faculty of Nursing Sciences, Université Laval, Quebec, Canada; 4 University of Sciences, Techniques, and Technology of Bamako, Bamako, Mali

**Keywords:** Adolescent, early sexuality, socioecological approach, phenomenological qualitative study, Benin

## Abstract

**Introduction:**

early adolescent sexuality is associated with an increase in risky sexual behaviour, unwanted pregnancies, and the occurrence of sexually transmitted infections. However, despite the efforts of governments and their partners, the implementation and effectiveness of appropriate and adapted services to improve adolescent sexual and reproductive health are lagging. Therefore, this study aimed to document determinants of early adolescent sexuality in the central district of Tchaourou in Benin based on a socio-ecological approach.

**Methods:**

an explorative and descriptive qualitative study was conducted using focus groups and individual interviews based on the socio-ecological model. Participants included adolescents, parents, teachers, and community leaders in Tchaourou.

**Results:**

the number of participants in each focus group was 8 (32). There were 20 girls and 12 boys aged 10-19 years, of whom 16 were students (7 females and nine males) and 16 were apprentice dressmakers and hairdressers. In addition, five participants attended individual interviews (two community leaders, one religious' leader, one teacher and one parent). Four themes were identified that influence early sexuality among adolescents and grouped into individual determinants related to knowledge about early sexuality; interpersonal determinants related to adolescents' function, including the influence of family and peers; community and organizational determinants related to where harmful sociocultural norms; political determinants comprising the disadvantaged socioeconomic status of the communities where adolescents live.

**Conclusion:**

many factors at multiple social levels influence early adolescent sexuality in the commune of Tchaourou in Benin. Therefore, interventions directed at these various levels are needed urgently.

## Introduction

Adolescence is a period during which the human being passes from the stage of the first appearance of secondary sexual characteristics to sexual maturity [1]. The adolescent acquires psychological structures and aptitudes that transform the child into an adult [2]. A transition is made from total social and economic dependence to relative independence [3]. Adolescent sexuality remains a concern worldwide. It is a social and medical issue. It is the product of a complex relationship between personal experiences, external influences, and social or moral contingencies [4]. In Africa, sexuality is communally taboo and involves sociocultural and socioeconomic contexts. Sexuality relates to the body, impressions, exchanges, love, procreation, and descent [5]. Although girls and boys experience this event at approximately the same age, the partner's characteristics and the expectations of young people attest to an experience still strongly gendered [6]. In addition, adolescents face several sexual and reproductive health (SRH) issues [7]. Several studies have shown that adolescents have their first sexual experiences while still in school [8-11]. In Africa, the age at first sex (AFS), the average age adolescents become sexually active, varies depending on the country. In Benin, according to the 2017-2018 Demographic and health survey (DHS) data, women have their first sexual intercourse earlier than men. Among young women aged 15-19, 12% had already started their sexual life before reaching 15, compared to 6% among young men aged 15-19 [12]. In the commune of Tchaourou, the median age at first sexual intercourse among women aged 15-19 is estimated at 16.64 years [13]. Moreover, the high proportion of unwanted births 58.9% among 15-19 demonstrates that adolescent sexual and reproductive health problems remain a reality in Tchaourou [14]. Civil society organizations play an increasingly important role in promoting adolescent reproductive health by promoting health education activities, developing community or clinical services, or advocating [15-17]. However, few evaluations of community-based adolescent and youth sexual and reproductive health programs are conducted in Africa, particularly in Benin [18]. In Benin, several interventions have been made for adolescents to prevent and fight against early sexuality. For example, in the commune of Tchaourou, non-governmental organization (NGOs) and associations implement interventions that target mainly adolescents and young people. However, there is a lack of evidence about the sexuality of adolescents in this commune. Therefore, this study aimed to document determinants of early adolescent sexuality in the central district of Tchaourou based on a socio-ecological approach.

## Methods

We followed Consolidated Criteria for Reporting Qualitative (COREQ) guidelines [19] when writing this manuscript.

**Study setting:** the commune of Tchaourou is in the department of Borgou and covers an area of 7,256 km^2^. It represents about 28% of the total area of this department and about 6.5% of the national territory. It is the largest commune in Benin in terms of size. The commune is subdivided into seven arrondissements and is organized into 36 villages and districts comprising several localities.

**Study design:** we conducted an exploratory and descriptive qualitative study using semi-structured interviews and focus groups. Qualitative research allows the understanding of social phenomena in natural contexts by focusing on all participants' meanings, experiences, and perspectives [20].

**Sampling and study participants:** participants were selected based on purposive sampling by maximum variation. To be included in the study, participants had to be adolescents willing to participate, parents, teachers, or religious or community leaders who had lived in the locality for a long time.

**Recruitment:** the mediator of a local NGO served as a key informant and recruited the participants. Once recruited, participants were informed about the purpose and procedure of the study. Verbal informed consent was obtained, and each participant signed the form. All were informed that they could withdraw from the study at any time.

**Data collection:** data were collected in January 2022 through focus groups (n= 4) with adolescents and teachers and semi-individual structured interviews with parents and leaders (n = 5). For each category of participant, an interview guide was developed and pretested. The interview guides included open questions about participants' knowledge and factors influencing early sexuality. The interviews were in French. Therefore, it was pretested and adjusted accordingly. Interviews took place in a social centre for adolescents learning a trade, at school for students and teachers, and in the community for parents and leaders. The interviews were recorded with the consent of the participants and between 25 and 35 minutes.

**Data analysis:** the recorded interviews were transcribed in verbatim form. They were checked for compliance and accuracy. Finally, a thematic analysis of the data using QDA Miner software was carried out. The stages of thematic analysis, as proposed by Braun and Clarke (2006), included the familiarization with the data, assigning preliminary codes to the data to describe its content, searching for patterns or themes in the codes across the different interviews, reviewing the themes, defining and naming the themes and producing the analysis report [21].

**Ethical considerations:** the study was approved by Benin's National Ethics and Health Research Committee (Ethics notice number 49 of 1 December 2021). In addition, the mayor of the commune of Tchaourou has permitted the study to be conducted.

## Results

The number of participants in each FG was 8 (32). There were 20 girls and 12 boys aged 10 - 19 years, of whom 16 were students (7 females and nine males) and 16 were apprentice dressmakers and hairdressers (Table 1). In addition, five participants attended individual interviews (two community leaders, one religious' leader, one teacher, and one parent) Table 2.

**Table 1:** socio-demographic characteristics of adolescents

Focus group	Age (years)	Occupation	Participants (n)
G1	10 -19	Seamstress apprentice	5 females ; 3 males (n = 8)
G2		Students	4 females; 4 males (n = 8)
G3		Students	3 females; 5 males (n = 8)
G4		Apprentice hairdresser	8 females (n = 8)

**Table 2:** socio-demographic characteristics of leaders, parents, and teachers

IDI	Age (years)	Profession
Community leader (CL1)	76	Village leader
Community leader (CL2)	72	Neighbourhood leader
Religious leader (RL)	65	Pastor
Parent (P)	56	Retired
Teacher (T)	32	Biology teacher

**Socio-ecological determinants of adolescents' early sexuality in Tchaourou, Benin:** participants reported factors influencing early adolescent sexuality. Emerged elements are grouped into five (5) categories: individual, interpersonal, organizational, community, and political determinants.

**Individual determinants:** all participants know early sexuality but define it according to their perception and understanding. At first glance, the analysis of the participants' interviews reveals no distinct definition of early sexuality. However, most respondents would agree that it is a common phenomenon. Based on what was said, early sexuality is not a foreign topic to the participants. This allows us to conclude that there is a perfect knowledge of early sexuality. The following statements evidence this: “We talk about early sexuality when the first sexual intercourse is rapid; when one starts sexuality without reaching adulthood” (G1, G2, G3, G4). Concerning the age of first sexual experience, our analysis shows that more than half of the adolescents interviewed are already sexually active. In addition, the age for some adolescents' sexual experiences ranged from 13 to 17, depending on the group of adolescents. For our participants, the ideal time to have sexual intercourse varies according to the knowledge and perception of each adolescent. The teenager is searching for his identity and no longer respects this ideal moment. Thus, the answers are multiple and varied 20 years old; 18 years old; 19 years old; 15 years old; 25 years old; after school; after graduation.

**Interpersonal and organizational determinants:** the community in which adolescents live has a significant impact on their sexuality. Most participants stated that very few parents discuss sexuality with their children because it is taboo. Thus, the lack of parent-child communication on sexuality during our interviews was considered a strategy to discourage adolescents from engaging in early sexual practices. Furthermore, some adolescents and teachers believe that parents cause this early sexuality. On the other hand, one community leader feels shameful talking about sexuality with his child. For instance, he said: “It is a shame for some parents to talk about sex with their children” (CL2). Almost all respondents raised the influence of peers (lousy company) as a favourable gateway for early sexuality. The participants underlined the names of local structures working in the locality of Tchaourou in prevention and the fight against early sexuality: “The NGO associació d'estudis fallers (ADEF) and the youth center Amour et Vie of ABMS/PSI. These structures identified peer educators, raised awareness, and made home visits to educate adolescents. The involvement of these NGOs in the fight against early sexuality among adolescents in Tchaourou has considerably reduced the pregnancy rate in general education schools. They came to talk to us about sexuality and communicable diseases” (G1, G2, G3, G4). In general, the participants learned a lot about sexual and reproductive health. Therefore, the developed themes are used to reflect the community's reaction to the adolescents' need for information.

**Community and political determinants:** as a result, leaders, parents, and teachers' perceptions of adolescent sexuality question the effectiveness of interventions because of the culture and taboo around sexuality. “It is a socio-cultural problem, and it is linked to their tradition. The Fulani will tell you that to avoid shame, they must quickly throw the girl to a man so that she can manage her sexuality” (CL2) Access to sexual information is a critical issue in any community. Unfortunately, most adolescents do not turn to parents or teachers for sexual information needs. One interviewee reported: “They never came to my house, but we look for them to talk to them” (P). Information and communication technologies make it easier for adolescents to access information on sexuality. However, the influence of pornographic images on social networks comes into play in Tchaourou. Some community leaders and parents also mainly mention the effect of television. “I am talking about televisions, especially cell phones or at certain times, you do not know, and they take them behind you” (LC1, LR, P). Parents' poverty and socioeconomic status are factors that should be considered. In addition, the participants listed several factors as sources of these early sexual relationships. “The vulnerable situation of parents, whose lack of means leads some girls to engage in sexuality quickly” (RL).

## Discussion

The objective of this study was to document determinants of early adolescent sexuality in the central district of Tchaourou based on a socioecological approach. Our findings reveal that several determinants contribute to preventing and controlling early sexuality. These determinants can be found in different levels of the socioecological model, such as interpersonal determinants related to adolescents' function, including the influence of family and peers; community and organizational determinants related to where harmful socio-cultural norms; political determinants comprising the disadvantaged socioeconomic status of the communities where adolescents live. We found that participants have a better knowledge of early sexuality. However, a study conducted in the district of Tchaourou reveals that adolescents and young people's understanding of sexuality remains limited, given their sources of information (parents and friends) [14]. Therefore, interventions should improve the level of knowledge of the entire community. On the other hand, the lack of responsibility of some parents leads adolescents to be sexually active at a younger age [22]. In our study, the median age at first sexual intercourse was 13, and the average was 16. Over half of the adolescents reported having their first sexual experience before age 17. This indicates early sexuality. A study conducted in Tanzania reports that 12% of adolescents reported having their first sexual intercourse under 13 [23]. Parent/adolescent communication about sexuality is essential and seen by many authors as an effective way to encourage responsible sexual behaviour in adolescents [2,24,25].

The results of a study reported that insufficient parent-child communication about sexuality is related to various reasons, such as the persistence of taboos and not knowing what to tell them due to the lack of preparation of parents in terms of information and training to communicate about sexuality [26]. However, in many areas, such as Tchaourou, discussions about sexuality and dating between parents and adolescents are exceptional [27]. Thus, without information from the family unit, adolescents turn to other sources, such as social media and the internet. In addition, the internet is an important source where teenagers seek health information, including health promotion and disease prevention messages and practical details on conceiving a child or the human body and reproductive systems [28]. However, others suggest that the internet and social media platforms might also have negative health consequences due to a false belief in privacy leading to more provocative behaviour and discussion around sex [29]. Social media are the primary mediators through which adolescents try out their sexuality. Therefore, parental monitoring remains vital and can moderate adolescents' internet and social media access [29,30]. Peer influence is another important determinant of early adolescent sexuality and negatively affects prevention and control interventions. Other studies suggested that the lack of parental control could lead girls to indulge in early sexual activity under duress or peer influence [31,32]. This could have enormous consequences that are difficult to manage later. Indeed, family structure is important to adolescent girls' behaviour [33]. In rural areas, studies show that supporting traditional and religious leaders and community-based organizations represents a solid potential to facilitate adolescents' access to the information and services they need [34]. In addition, some sources, such as health centers, counselling centers, and NGOs, are more empowered to disseminate information about sexuality to youth [5].

Some NGOs have invested in preventing and controlling early adolescent sexuality in Tchaourou. Several themes have been developed during their intervention period. First, the involvement of these structures in the fight against early sexuality has enabled adolescents to adopt better behaviours. Participants have acquired much knowledge about sexual and reproductive health through these activities. In addition, during the sessions with the NGOs, the adolescents learned about the risks they face regarding sexuality, including sexually transmitted infections (STIs) and HIV/AIDS, unwanted early pregnancies, and induced abortions. In their study, the effects noted by Thato, Jenkins and Dusitsin (2008) include increased knowledge of STIs and HIV/AIDS, increased awareness of pregnancy risks, and increased intention to use condoms [35]. The perception of leaders, parents, and teachers reflects the need to implement a sex education system for adolescents in Tchaourou. They believe that, according to parents, children should learn about sexuality at a reasonable age (18-21 years). It allows the child to enter adulthood. This is why it is good to delay the discussion time [5]. In the community of Tchaourou, sexuality appears to be taboo. Inside the family, parent-child communication, topics on sexuality, and intimate life remain unfruitful or silent because of sociological and cultural constraints in the communities studied. In these communities, sex is taboo and cannot be discussed publicly and with children [24,36]. Most adolescents live under an economic dependence on their parents. This situation can impact their level of knowledge about sexual risk prevention depending on whether they live in favorable or unfavorable economic conditions [2]. Conversely, the institutional approach assumes that information in improving sexual risk knowledge depends on political agendas and decision-makers importance to sexual and reproductive health [37]. The rationale for considering institutional factors in sexual activity is that some programs and services benefit young people and contribute to their prevention and risk reduction. The institutional approach appears to be a local action device to a global action device. This action can lead to behaviour changes at the individual level and then at the collective level [5]. In addition, difficult living conditions marked by poverty may push adolescents to engage in sexual relations early. However, interventions such as social cash transfers have been shown to delay the age of first sexual intercourse among adolescents while improving the economic situation of families [38].

**Limitations:** this study has limitations that are important to consider. Firstly, we could not observe the field as planned in our data collection tools. Participation in interviews and focus groups allowed for more exploratory and descriptive analysis. In addition to the preliminary steps taken, we could not meet with certain actors, particularly the district chief and the councillors. Their assessments of the effectiveness of these interventions and suggestions for improving the interventions in Tchaourou are essential

## Conclusion

From a phenomenological perspective, this study focused on determinants of early adolescent sexuality in the central district of Tchaourou based on a socioecological approach. Our results highlight that many factors at multiple social levels influence early adolescent sexuality in the commune of Tchaourou in Benin. Therefore, interventions directed at these multiple levels are needed urgently.

### What is known about this topic

- *Early adolescent sexuality is associated with an increase in risky sexual behaviour, unwanted pregnancies, and the occurrence of sexually transmitted infections;*
- *In Africa, sexuality is communally taboo and involves socio-cultural and socioeconomic contexts*.

### What this study adds

- *Many factors at multiple social levels influence early adolescent sexuality in the commune of Tchaourou in Benin;*
- *Harmful socio-cultural norms were reported to influence adolescents' sexual behaviours*.
